# Invasive *Galega officinalis* (Goat's rue) plants in Canada form a symbiotic association with strains of *Neorhizobium galegae* sv. officinalis originating from the Old World

**DOI:** 10.1002/ece3.5266

**Published:** 2019-05-26

**Authors:** Eden S. P. Bromfield, Sylvie Cloutier, Catherine Robidas, Thu Van Tran Thi, Stephen J. Darbyshire

**Affiliations:** ^1^ Ottawa Research and Development Centre Agriculture and Agri‐Food Canada Ottawa Ontario Canada

**Keywords:** Canada, *Galega officinalis* (Goat's rue), invasive, *Neorhizobium galegae* sv. officinalis

## Abstract

The toxic legume plant, *Galega officinalis,* is native to the Eastern Mediterranean and Black Sea regions. This legume is considered to be a noxious weed, and its establishment in Canada may have resulted from ornamental planting and/or field trials. In its native range, a highly specific nitrogen‐fixing symbiosis with the bacterium, *Neorhizobium galegae* symbiovar (sv.) officinalis, is required for normal growth. In North America, nothing is known about the bacterial symbionts of *G. officinalis*. Our purpose was to determine the species and symbiovar identity of symbiotic bacteria associated with invasive plants of *G. officinalis* at five sites in the province of Ontario, Canada.  Sequence analysis of four housekeeping (16S rRNA, *atp*D, *gln*II, and *rec*A) and two symbiosis (*nod*C and *nif*H) genes showed that all 50 bacterial isolates from root nodules of *G. officinalis* at the five Canadian sites were identical to strains of *N. galegae* sv. officinalis originating either from Europe or the Caucasus. Plant tests indicated that soils collected from four Canadian sites without a history of agriculture or presence of *G. officinalis* were deficient in symbiotic bacteria capable of eliciting nodules on this plant. Collectively our data support the hypothesis of anthropogenic co‐introduction of *G. officinalis* and its specific symbiotic bacterium into Canada from the Old World. Factors that may limit the spread of *G. officinalis* in new environments are discussed.

## 1 INTRODUCTION

*Galega officinalis* (Goat's rue) is a toxic leguminous plant that is considered to have originated in the Eastern Mediterranean and Black Sea regions but has been spread by anthropogenic means to Europe, Western Asia, and North Africa, and more recently to North and South America and New Zealand (CABI, 2019; Osterman et al., 2011; USDA‐ARS, 2019). While *G. officinalis* has a long history of ornamental and medicinal uses in Europe, it is usually considered a noxious weed because of its invasive characteristics and potential for poisoning livestock (CABI, 2019; CFIA, 2017; Chiej, 1984; EPPO, 2002; Tingey, 1971; USDA‐APHIS, 2010). Since its first introduction to North America in the late 19th Century as an ornamental or for evaluation in field trials (Bailey & Bailey, 1976; CABI, 2019; CFIA, 2017; Macoun, 1908; Tingey, 1971), *G. officinalis* has become established at several locations in Canada (Figure S1).

The rhizobia are root‐nodule bacteria that fix atmospheric nitrogen in symbiotic association with leguminous plants thereby supplying the host with nitrogen compounds necessary for growth. *Galega officinalis* and its non‐noxious relative, *G. orientalis* (fodder galega), form a highly specific symbiotic association with root‐nodule bacteria belonging to the species, *Neorhizobium galegae* (Andrews & Andrews, 2017; Lindstrom, 1989; Mousavi, Willems, Nesme, Lajudie, & Lindstrôm, 2015). No species other than *N. galagae* has been reported to induce root nodules on *Galega* plants (Österman et al., 2014).

*Neorhizobium galagae* is divided into two symbiovars (symbiotic varieties), officinalis and orientalis, that are capable of eliciting nodules on roots of *G. officinalis* and *G. orientalis,* but fix nitrogen only in association with the respective host plant species (Österman et al., 2014; Österman, Mousavi, Koskinen, Paulin, & Lindström, 2015; Radeva et al., 2001).

In preliminary surveys, we identified five sites in the province of Ontario, Canada, harboring established plants of *G. officinalis* that were nodulated by symbiotic bacteria.

Our purpose was to determine the species and symbiovar identity of the root‐nodule bacteria associated with plants of *G. officinalis* established at these sites. Bacterial identification was achieved by phylogenetic analysis of four housekeeping and two symbiosis gene sequences.

To determine whether bacteria capable of symbiosis with *G. officinalis* occur naturally in Canadian soils, we carried out plant infection tests using soils collected from several sites that were without a history of agriculture or *G. officinalis*.

## 2 MATERIALS AND METHODS

Five plants of *G. officinalis* were dug up at random from each four sites (S1–S4) in a 50 km radius of Ottawa as well as from a single site (S5) about 800 km distant in Sault Ste. Marie, Ontario; site descriptions and coordinates are given in Table 1.

**Table 1:** Site descriptions

Site	Location	Coordinates	Soil texture	Soil pH (1:1, soil:water)
(a) Sites with *Galega officinalis* plants				
S1	Ottawa, Ontario	45°27′10″N, 75°35′19″W	Silty clay	7.4
S2	Ottawa, Ontario	45°27′44″N, 75°37′32″W	Silty clay loam	7.5
S3	Ottawa, Ontario	45°26′47″N, 75°39′9″W	Sandy loam	7.7
S4	Ottawa, Ontario	45°30′10″N, 75°29′50″W	Fine loamy sand	7.8
S5	Sault Ste. Marie, Ontario	46°30′59″N, 84°20′40″W	Silty loam	7.8
(b) Sites without a history of agriculture or *Galega officinalis*				
S6	Quyon, Québec	45°29′23″N, 76°23′18″W	Silty loam	7.3
S7	Carp, Ontario	45°15′59″N, 76°08′52″W	Medium to fine loamy sand	7.2
S8	Carp, Ontario	45°15′13″N, 76°08′24″W	Fine sandy loam	7.1
S9	Kemptville, Ontario	45°03′49″N, 75°49′20″W	Loam	7.4

Root nodules were collected from tap and lateral roots of the sampled plants and stored at 4°C in vials containing anhydrous silica gel (Date & Halliday, 1987). Bacteria were isolated from surface sterilized nodules, grown at 28°C on yeast extract mannitol (YEM) agar medium (Tang, Bromfield, Rodrigue, Cloutier, & Tambong, 2012) and purified by repeated streaking and single‐colony picking. Pure bacterial cultures were maintained at −80°C in 20% w/v glycerol.

The 50 bacterial isolates that were analyzed in this study are listed in Table S1; reference taxa are shown in Table S2.

Preparation of bacterial genomic DNA was as described by Tang et al., 2012. Amplification and sequencing of housekeeping (16S rRNA, *atp*D, *gln*II, and *rec*A) and symbiosis (*nif*H [nitrogen fixation] and *nod*C [nodulation]) genes were carried out using primers and conditions described in Table S3. As suitable primers for amplification and sequencing of the *nod*C and *nif*H genes of *N. galagae* were not available in the literature, we designed primers based on the full genome sequence of *N. galegae* HAMBI 1141 sv. officinalis (GenBank accession no. HG938357) using Geneious Software (Biomatters Inc., USA) (Table S3). GenBank accession numbers of the nucleotide sequences used in this work are given in Tables S1 and S2.

Bayesian phylogenetic trees were inferred using MrBayes version 3.2.1 (Altekar, Dwarkadas, Huelsenbeck, & Ronquist, 2004) with default priors as previously described (Yu, Cloutier, Tambong, & Bromfield, 2014); Maximum Likelihood (ML) trees (Guindon et al., 2010) were inferred as detailed by Tang et al. (2012) using 1,000 nonparametric bootstrap replications to assess support. Best‐fit substitution models were selected using the Bayesian information criterion implemented in jModelTest version 2 (Darriba, Taboada, Doallo, & Posada, 2012). As tree topologies from Bayesian and ML analyses were similar, only the Bayesian trees are shown in this work.

To determine whether symbiotic bacteria capable of eliciting nodules on *G. officinalis* occur naturally in Canada, soil samples were collected from four sites (S6–S9, within a 150 km radius of Ottawa, Ontario) that were without a history of agriculture or *G. officinalis* (Table 1). Thirty random soil samples from each site were collected to a depth of 15 cm with aseptic precautions; soil samples were pooled to form a composite sample for each site. Seeds collected from plants of *G. officinalis* growing in Ottawa, Ontario, were surface sterilized, grown in axenic Leonard jar assemblies supplied with nitrogen‐free nutrient solution (Tang et al., 2012) and inoculated with soil suspensions (1:1, soil:water). Plants were maintained in a temperature‐controlled growth chamber for 35 days using conditions described by Bromfield et al. (2010). Controls consisted of uninoculated plants and plants inoculated with HAMBI 1141, a nitrogen‐fixing reference strain of *N. galegae* sv. officinalis and with a bacterial isolate (G122) from *Galega* plants growing at site S2 (Ottawa).

## 3 RESULTS AND DISCUSSION

*Galega officinalis* plants sampled from the five Canadian sites (S1 to S5) were vigorous, showed no signs of nitrogen deficiency, and were extensively nodulated by symbiotic bacteria (Figure S2). Effective nitrogen fixation by bacterial symbionts was indicated by the presence of leghemoglobin (a red colored hemoprotein required for nitrogen fixation), in the interior of the nodules. All five of these sites had moist soils with pH values above 7.0 (Table 1) and showed evidence of significant anthropogenic disturbance and import of soil material. This suggests that the *G. officinalis* plants at these sites were probably introduced along with imported soil material.

Isolates of symbiotic bacteria from nodules of *G. officinalis* at Canadian sites produced colonies on YEM agar medium after 7 days at 28°C that were beige, round, convex, ca. 1–1.5 mm diameter and similar to those of *N. galagae* reference strains, HAMBI 540^T^ (sv. orientalis) and HAMBI 1141 (sv. officinalis).

**Figure 1:**
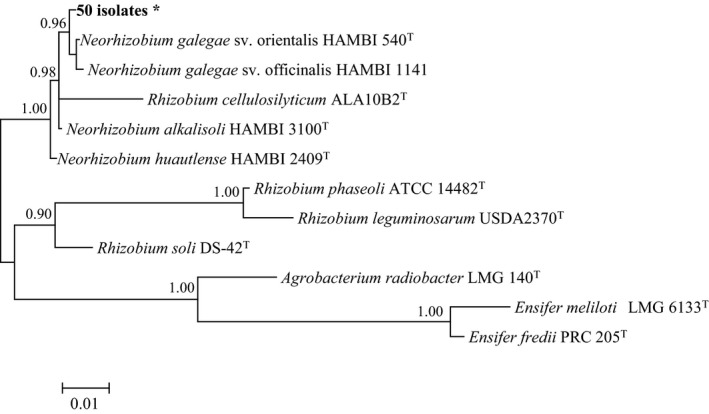
Bayesian phylogenetic tree of 16S rRNA gene sequences of reference taxa and 50 bacterial isolates from *Galega officinalis* plants at five Canadian sites (HKY + I substitution model). Only posterior probabilities >90% are shown. Scale bar represents expected number of substitutions per site

As was expected based on the high specificity of the *Galega*–bacterial symbiosis (Österman et al., 2014, 2015; Radeva et al., 2001), analysis of almost full‐length 16S rRNA gene sequences (1,400 bp) indicated that all 50 bacterial isolates from sites S1 to S5 belonged to the genus *Neorhizobium* as they were placed in a phylogenetic cluster with HAMBI 540^T^, the type strain of the species, *N. galegae* (Figure 1).

Further analyses based on BLASTn database searches revealed that each of the 50 isolates had *atp*D, *gln*II, and *rec*A gene sequences that were identical to one of four *N. galegae* sv. officinalis strains: HAMBI 1183 (=G6 = IAM14208) and HAMBI 1186 (=G10) probably originating from Kew Gardens, United Kingdom (K. Lindström and P. Oivanen, University of Helsinki, Finland, personal communication; Lindstrom, 1989); HAMBI 2425 (=IT1) originating from Italy (Terefework, Kaijalainen, & Lindström, 2001); and HAMBI 2544 (=G032) originating from the Russian Caucasus (Andronov et al., 2003).

**Figure 2:**
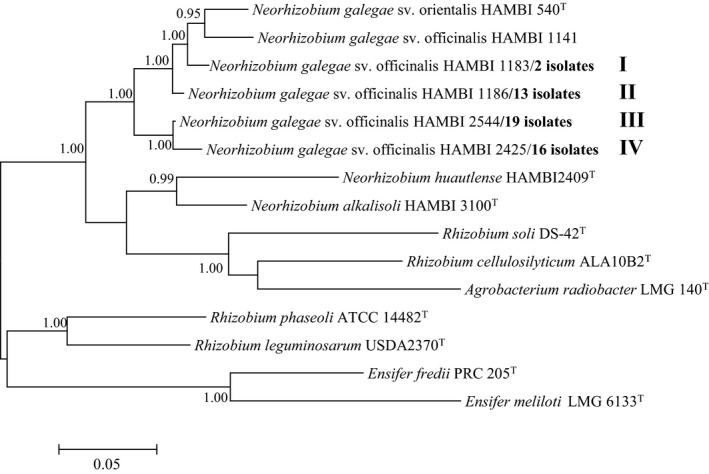
Bayesian phylogenetic tree (GTR + G + I substitution model) of *atp*D–*gln*II–*rec*A concatenated housekeeping gene sequences (1,383 bp) for reference taxa and 50 bacterial isolates from *Galega officinalis* plants at five Canadian sites. Roman numerals in bold type refer to lineage numbers. Posterior probabilities >90% are shown. Bar, expected substitutions per site

A phylogenetic tree (Figure 2) of concatenated *atp*D–*gln*II–*rec*A housekeeping gene sequences (1,383 bp) confirmed that the 50 bacterial isolates were divided into four lineages (multiple locus genotypes) each represented by a strain of *N. galegae* sv. officinalis. The identification of symbiovars in root‐nodule bacteria is based on differences in symbiosis gene sequences (Rogel, Ormeño‐Orrillo, & Romero, 2011). In this connection, BLASTn database searches confirmed that all 50 isolates of *N. galegae* belong to symbiovar officinalis as they possess *nod*C (795 bp) and *nif*H (650 bp) symbiosis gene sequences that are at least 99.9% similar to *N. galegae* sv. officinalis HAMBI 1141.

Data for the frequency of bacterial strains at sites S1–S5 (Table 2) show that at least three of the four strains were detected at each of the Ottawa sites (S1–S4, separated by distances of between 5 and 50 km) whereas at the geographically distant Sault Ste. Marie site (S5, about 800 km from Ottawa) only one strain was encountered. These differences in the relative occurrence of *N. galegae* sv. officinalis strains between geographically disparate locations may reflect the import of *G. officinalis* plant material and associated bacterial symbiont into Canada from different sources, or other factors such as sampling error and genetic drift.

**Table 2:** Frequency of strains of *Neorhizobium galegae* sv. officinalis at five Canadian sites based on sequence analysis of *atp*D, *gln*II, and *rec*A genes

Lineage^a^	Strain^b^	Bacterial isolates from					Total isolates
		Ottawa				Sault Ste. Marie	
		Site S1	Site S2	Site S3	Site S4	Site S5	
I	HAMBI 1183	–	2	–	–	–	2
II	HAMBI 1186	5	4	3	1	–	13
III	HAMBI 2544	4	3	4	8	–	19
IV	HAMBI 2425	1	1	3	1	10	16

Note

Data are based on 10 bacterial isolates from each site.

a As defined in Figure 2.

b Strains of *Neorhizobium galegae* sv. officinalis originating either from Europe or the Caucasus.

In plant tests, negative control plants and plants inoculated with suspensions of soil collected from four sites (S6–S9) without a history of *G. officinalis* lacked root nodules. These plants had small yellow shoots due to the absence of symbiotic nitrogen fixation. In contrast, positive control plants inoculated with *N. galegae* sv. officinalis HAMBI 1141 and isolate G122 from site S2 (Ottawa) possessed large dark green shoots and numerous nitrogen‐fixing root nodules. These results indicate that bacterial symbionts of *G. officinalis* are absent from Canadian soils where the plant host does not occur.

Collectively our data support the hypothesis of anthropogenic co‐introduction of *G. officinalis* and its specific bacterial symbiont into Canada probably from parts of Europe or the Caucasus. The transport of bacteria either on seed or in soil containing plant material is a possible mechanism that could account for the co‐introduction of *G. officinalis* and its specific symbiont to Canada.

The fact that symbiotic *G. officinalis* plants were only found at Canadian sites with soils above pH 7.0 (range 7.4–7.8; Table 1) is consistent with reports from the United States (Oldham & Ransom, 2009) and Spain (González‐Andrés, Redondo, Pescador, & Urbano, 2004) of plants (presumably symbiotic) growing in soils with pH ranges of 7.3–7.5 and 7.7–8.2, respectively. This suggests that the apparent adaptation of *G. officinalis* to soils above pH 7.0 together with its high level of symbiotic specificity may serve as important factors limiting the spread of the plant in new environments where the specific nitrogen‐fixing bacterial symbiont (*N. galegae* sv. officinalis) is absent. This is consistent with the observation that *G. officinalis* is established at only a few localized sites in Canada (Figure S1) despite having been cultivated as early as 1897 (Bailey & Bailey, 1976; Macoun, 1908) and herbarium records showing that the plant was grown in gardens throughout the first half of the 20th Century.

## CONFLICT OF INTEREST

The authors declare that there are no conflicts of interest.

## AUTHOR CONTRIBUTIONS

ESPB, SC, and SJD conceived the experiments. CR, SC, ESPB, SJD, and TVTT performed the experiments; SC and ESPB analyzed the data. ESPB drafted the manuscript. All authors read, contributed to, and approved the final manuscript.

## Data Availability

Nucleotide sequences of bacterial isolates from *G. officinalis* plants in Canada were deposited in DDBJ/GenBank, under Accession Numbers: KT869496‐KT869545 (16S rRNA), KT869546‐KT869595 (*atp*D), KT869596‐KT869645 (*gln*II), KT869646‐KT869695 (*rec*A), KT869696‐KT869745 (*nif*H), and KT869746‐KT869795 (*nod*C). Voucher specimens of *Galega officinalis* plants collected at Canadian sites S1–S5 are available at the Vascular Plant Herbarium (DAO), Agriculture and Agri‐Food Canada, 960 Carling Ave., Ottawa, Ontario, K1A 0C6, Canada. http://www.agr.gc.ca/eng/science-and-innovation/agriculture-and-agri-food-research-centres-and-collections/national-collection-of-vascular-plants-dao. All other data are presented in the Supporting Information.
