# Supplementary material for: Invasive Galega officinalis (Goat's rue) plants in Canada form a symbiotic association with strains of Neorhizobium galegae sv. officinalis originating from the Old World
Source: Ecol Evol. 2019 May 26;9(12):6999–7004. doi: 10.1002/ece3.5266 (PMC6662265; doi:10.1002/ece3.5266)
Supplement: Supplementary file 1 [file ECE3-9-6999-s001.pdf]

Table S1. GenBank accession numbers for housekeeping (16S rRNA, *atp*D, *gln*II, and *rec*A) and symbiosis (*nif*H and *nod*C) gene sequences of 50 bacterial isolates from *Galega officinalis* plants at five Canadian sites.

Lineage[a]	Isolate	Site	16S rRNA	*atp*D	*gln*II	*rec*A	*nif*H	*nod*C
II	G1	S1	KT869496	KT869546	KT869596	KT869646	KT869696	KT869746
III	G3	S1	KT869497	KT869547	KT869597	KT869647	KT869697	KT869747
II	G18	S1	KT869498	KT869548	KT869598	KT869648	KT869698	KT869748
II	G25	S1	KT869499	KT869549	KT869599	KT869649	KT869699	KT869749
II	G34	S1	KT869500	KT869550	KT869600	KT869650	KT869700	KT869750
II	G35	S1	KT869501	KT869551	KT869601	KT869651	KT869701	KT869751
III	G46	S1	KT869502	KT869552	KT869602	KT869652	KT869702	KT869752
IV	G49	S1	KT869503	KT869553	KT869603	KT869653	KT869703	KT869753
III	G66	S1	KT869504	KT869554	KT869604	KT869654	KT869704	KT869754
III	G67	S1	KT869505	KT869555	KT869605	KT869655	KT869705	KT869755
III	G81	S2	KT869506	KT869556	KT869606	KT869656	KT869706	KT869756
III	G83	S2	KT869507	KT869557	KT869607	KT869657	KT869707	KT869757
I	G91	S2	KT869508	KT869558	KT869608	KT869658	KT869708	KT869758
II	G93	S2	KT869509	KT869559	KT869609	KT869659	KT869709	KT869759
II	G106	S2	KT869510	KT869560	KT869610	KT869660	KT869710	KT869760
II	G107	S2	KT869511	KT869561	KT869611	KT869661	KT869711	KT869761
II	G121	S2	KT869512	KT869562	KT869612	KT869662	KT869712	KT869762
III	G122	S2	KT869513	KT869563	KT869613	KT869663	KT869713	KT869763
IV	G136	S2	KT869514	KT869564	KT869614	KT869664	KT869714	KT869764
I	G138	S2	KT869515	KT869565	KT869615	KT869665	KT869715	KT869765
III	G153	S3	KT869516	KT869566	KT869616	KT869666	KT869716	KT869766
II	G156	S3	KT869517	KT869567	KT869617	KT869667	KT869717	KT869767
II	G166	S3	KT869518	KT869568	KT869618	KT869668	KT869718	KT869768
II	G168	S3	KT869519	KT869569	KT869619	KT869669	KT869719	KT869769
III	G181	S3	KT869520	KT869570	KT869620	KT869670	KT869720	KT869770
III	G182	S3	KT869521	KT869571	KT869621	KT869671	KT869721	KT869771
IV	G196	S3	KT869522	KT869572	KT869622	KT869672	KT869722	KT869772
IV	G197	S3	KT869523	KT869573	KT869623	KT869673	KT869723	KT869773
III	G211	S3	KT869524	KT869574	KT869624	KT869674	KT869724	KT869774
IV	G212	S3	KT869525	KT869575	KT869625	KT869675	KT869725	KT869775
III	G226	S4	KT869526	KT869576	KT869626	KT869676	KT869726	KT869776
III	G227	S4	KT869527	KT869577	KT869627	KT869677	KT869727	KT869777
III	G241	S4	KT869528	KT869578	KT869628	KT869678	KT869728	KT869778
III	G242	S4	KT869529	KT869579	KT869629	KT869679	KT869729	KT869779
IV	G243	S4	KT869530	KT869580	KT869630	KT869680	KT869730	KT869780
III	G256	S4	KT869531	KT869581	KT869631	KT869681	KT869731	KT869781
III	G257	S4	KT869532	KT869582	KT869632	KT869682	KT869732	KT869782
II	G272	S4	KT869533	KT869583	KT869633	KT869683	KT869733	KT869783
III	G286	S4	KT869534	KT869584	KT869634	KT869684	KT869734	KT869784
III	G287	S4	KT869535	KT869585	KT869635	KT869685	KT869735	KT869785
IV	G301	S5	KT869536	KT869586	KT869636	KT869686	KT869736	KT869786
IV	G302	S5	KT869537	KT869587	KT869637	KT869687	KT869737	KT869787
IV	G306	S5	KT869538	KT869588	KT869638	KT869688	KT869738	KT869788
IV	G307	S5	KT869539	KT869589	KT869639	KT869689	KT869739	KT869789
IV	G311	S5	KT869540	KT869590	KT869640	KT869690	KT869740	KT869790
IV	G312	S5	KT869541	KT869591	KT869641	KT869691	KT869741	KT869791
IV	G316	S5	KT869542	KT869592	KT869642	KT869692	KT869742	KT869792
IV	G317	S5	KT869543	KT869593	KT869643	KT869693	KT869743	KT869793
IV	G321	S5	KT869544	KT869594	KT869644	KT869694	KT869744	KT869794
IV	G323	S5	KT869545	KT869595	KT869645	KT869695	KT869745	KT869795

[a]Lineage as defined in Figure 2.

Table S2. GenBank accession numbers of 16S rRNA, *atp*D, *gln*II, and *rec*A gene sequences for reference taxa.

Reference strain	16S rRNA	*atp*D	*gln*II	*rec*A
Neorhizobium galegae sv. orientalis HAMBI 540^T	AB680726	KF206641	KF206809	KF206896
Neorhizobium galegae sv. officinalis HAMBI 1141	HG938355	KF206640	KF206808	KF206895
Neorhizobium galegae sv. officinalis HAMBI 1186	n/a	KF206557	KF206729	KF206813
Neorhizobium galegae sv. officinalis HAMBI 1183	n/a	KF206556	KF206728	KF206812
Neorhizobium galegae sv. officinalis HAMBI 2544	n/a	FK206579	KF206751	KF206835
Neorhizobium galegae sv. officinalis HAMBI 2425	n/a	KF206570	KF206741	KF206826
Neorhizobium alkalisoli HAMBI 3100^T	EU074168	KF206604	KF206776	FK206860
Neorhizobium huautlense HAMBI 2409^T	NR_024863	KF206569	KF206740	KF206825
Rhizobium cellulosilyticum ALA10B2^T	NR_043985	KF206617	KF206788	KF206873
Rhizobium phaseoli ATCC 14482^T	NR044112	EF113151	JN580716	KF206876
Rhizobium leguminosarum USDA2370^T	U29386	AM418783	AF169586	AM182125
Rhizobium soli DS-42^T	EF363715	KF206629	KF206798	KF206885
Agrobacterium radiobacter LMG 140^T	AE007869	KF206563	KF206734	KF206818
Ensifer meliloti LMG 6133^T	D14509	AM418760	KR818748	AM182133
Ensifer fredii PRC 205^T	X67231	AJ294402	AF169591	AJ294379

n/a, sequences not available in databases

Table S3. Primers and DNA amplification conditions.

Primer[a]	Direction	Sequence 5'-3'	PCR cycling conditions[b]	Reference
16Sa	Forward	CGCTGGCGGCAGGCTTAACA	5 min 95ºC, 31x (45 sec 95ºC, 30 sec	van Berkum *et al.* 2000
16Sb	Reverse	CCAGCCGCAGGTTCCCCT	68ºC, 2 min 72ºC), 7 min 72ºC	van Berkum *et al.* 2000
16Sa2	Forward	GGCAGCAGTGGGGAATATTG	Used only for sequencing	Mantelin *et al.* 2006
16Sa3	Forward	CCTGGGGAGTACGGTCGCAAG	Used only for sequencing	Mantelin *et al.* 2006
16Sb2	Reverse	GGGACTTAACCCAACATCT	Used only for sequencing	Mantelin *et al.* 2006
16Sb3	Reverse	GGCACGAAGTTAGCCGGGGC	Used only for sequencing	Mantelin *et al.* 2006
atpD273f	Forward	SCT GGG SCG YAT CMT GAA CGT	5 min 95ºC, 32x (45 sec 95ºC, 30 sec	Gaunt *et al.* 2001
atpD771r	Reverse	GCC GAC ACT TCC GAA CCN GCC TG	70ºC, 1 min 72ºC), 7 min 72ºC	Gaunt *et al.* 2001
*gln*II12F	Forward	YAAGCTCGAGTACATYTGGCT	5 min 95ºC, 30x (45 sec 95ºC, 1min	Vinuesa *et al.* 2005
*gln*IITSr	Reverse	SGAGCCGTTCCAGTCGGTGTCG	15 sec 72ºC), 7 min 72ºC	Stepkowski *et al.* 2005
recA-91F	Forward	TTCGGTAAGGGMTCGATHATG	5 min 95ºC, 32x (45 sec 95ºC, 30 sec	Mousavi *et al*. 2014
recA-595R	Reverse	CGHATCTGGTTGATGAAGATNACCAT	65ºC, 30 sec 72ºC), 7 min 72ºC	Mousavi *et al.* 2014
nodC229F	Forward	TATGGCGGCAAGTTTCGAGT	5 min 95ºC, 34x (45 sec 95ºC, 30 sec	This study
nodC1264R	Reverse	GGGATTCGGAGGTCACGATC	62ºC, 1 min 72ºC), 7 min 72ºC	This study
nifH21F	Forward	CGCATTCTACGGAAAAGGCG	5 min 95 °C, 35x (45 sec 95°C, 30	This study
nifH792R	Reverse	TTCCATCGTGATCGGAGTCG	sec 62°C, 1 min 72°C), 7 min 72 °C	This study

[a], Primers used for PCR amplification and sequencing unless otherwise stated.
[b], PCR cycling conditions were modified in this study.

References

Gaunt, M., Turner, S., Rigottier-Gois, L., Lloyd-Macgilp, S. and Young, J. (2001). Phylogenies of atpD and recA support the small subunit rRNA-based classification of rhizobia. International Journal of Systematic and evolutionary microbiology, 51 (6), 2037-2048.

Mantelin, S., Saux M., Zakhia F., Béna G., Bonneau S., Jeder H., de Lajudie P., Cleyet-Marel J. (2006). Emended description of the genus Phyllobacterium and description of four novel species associated with plant roots: Phyllobacterium bourgognense sp. nov., Phyllobacterium ifriqiyense sp. nov., Phyllobacterium leguminum sp. nov. and Phyllobacterium brassicacearum sp. nov. International Journal of Systematic and Evolutionary Microbiology, 56(4), 827-839.

Mousavi, S., Österman, J., Wahlberg, N., Nesme, X., Lavire, C., Vial, L., Paulin, L., de Lajudie, P. and Lindström, K. (2014). Phylogeny of the Rhizobium–Allorhizobium–Agrobacterium clade supports the delineation of Neorhizobium gen. nov. Systematic and Applied Microbiology, 37(3), 208-215.

Stepkowski, T., Moulin, L., Krzyzanska, A., McInnes, A., Law, I. and Howieson, J. (2005). European Origin of Bradyrhizobium Populations Infecting Lupins and Serradella in Soils of Western Australia and South Africa. Applied and Environmental Microbiology, 71(11), 7041-7052.

van Berkum, P. and Fuhrmann, J. (2000). Evolutionary relationships among the soybean bradyrhizobia reconstructed from 16S rRNA gene and internally transcribed spacer region sequence divergence. International Journal of Systematic and Evolutionary Microbiology, 50(6), 2165-2172.

Vinuesa, P., Silva, C., Werner, D. and Martínez-Romero, E. (2005). Population genetics and phylogenetic inference in bacterial molecular systematics: the roles of migration and recombination in Bradyrhizobium species cohesion and delineation. Molecular Phylogenetics and Evolution, 34(1), 29-54.

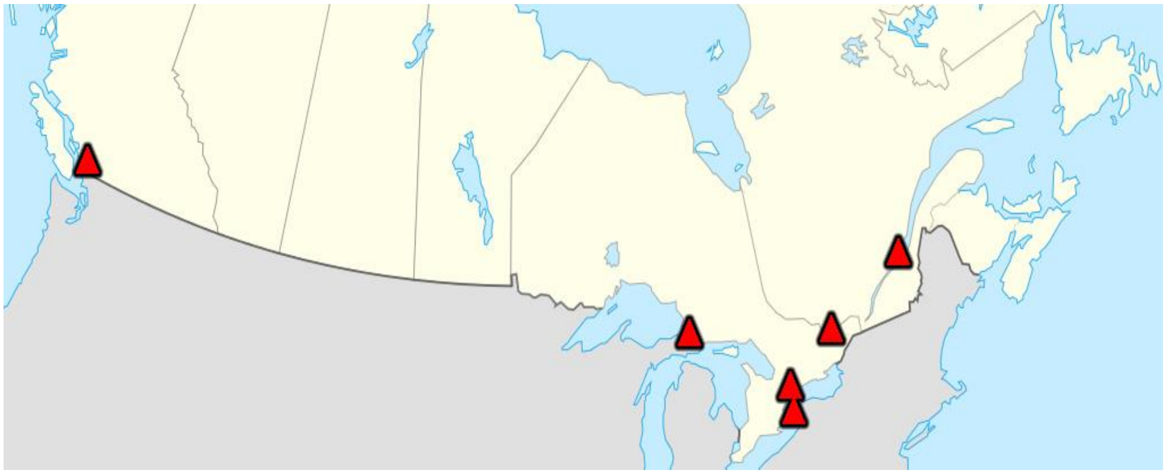

Fig. S1. Distribution of *Galega officinalis* (goat’s rue) in Canada based on data from herbarium specimens at Agriculture and Agri-Food Canada, Ottawa (DAO), University of British Columbia, Vancouver (UBC), and Université Laval, Québec (QFA).

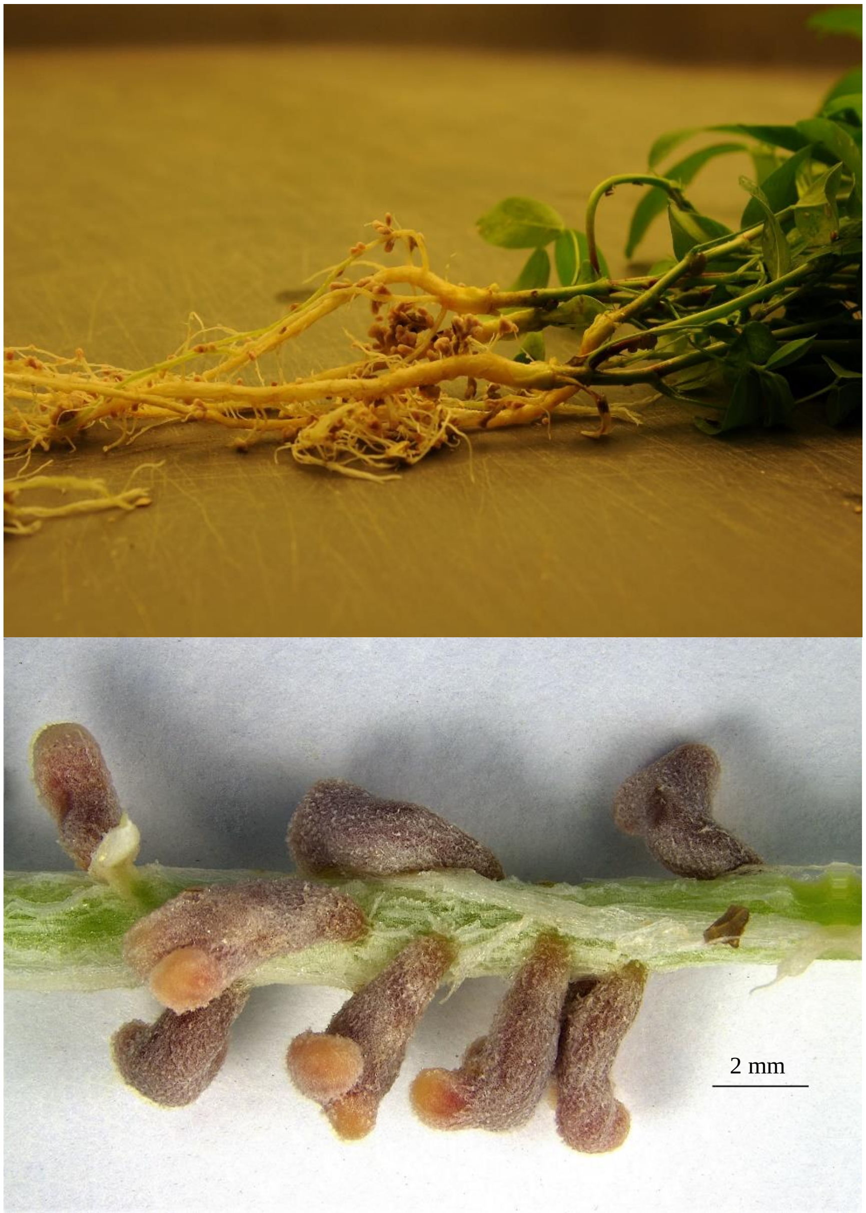

Fig. S2. Efficient nitrogen fixing nodules occupied by N*eorhizobium galegae* sv. officinalis on roots of *Galega officinalis*.
